# Sex Differences in Fecal Microbiota Correlation With Physiological and Biochemical Indices Associated With End-Stage Renal Disease Caused by Immunoglobulin a Nephropathy or Diabetes

**DOI:** 10.3389/fmicb.2021.752393

**Published:** 2021-11-26

**Authors:** Peng Zhang, Jiali Fang, Guanghui Li, Lei Zhang, Xingqiang Lai, Lu Xu, Luhao Liu, Yunyi Xiong, Li Li, Tao Zhang, Jiao Wan, Hailin Xu, Rongxin Chen, Weiting Zhang, Junjie Ma, Zheng Chen

**Affiliations:** Organ Transplant Center, The Second Affiliated Hospital of Guangzhou Medical University, Guangzhou, China

**Keywords:** end-stage renal disease, IgA nephropathy, type-2 diabetes mellitus, gut microbiota, clinical immune indicators, chronic kidney disease

## Abstract

This study investigated the sex-specific differences in the correlation between intestinal microbiota and end-stage renal disease. Here, we compared the differences in the gut microbiota of male and female healthy controls (HC) and patients with end-stage renal disease (ESRD) caused by immunoglobulin A (IgA) nephropathy (ESRD-IgAN) or type-2 diabetes mellitus (ESRD-T2DM) using high-throughput sequencing of the 16S rRNA gene. We also analyzed the correlation between gut microbiota and clinical immune indicators. We assigned 8, 10, 5, 7, 11, and 20 volunteers to female HC, ESRD-IgAN, and ESRD-T2DM, and male HC, ESRD-IgAN, and ESRD-T2DM, respectively. The results showed sex-specific differences in both physiological and biochemical indices and intestinal microbiota composition, as well as the correlation between them. The correlations between physiological and biochemical indices in men were significantly lower than those in women, especially for indices related to immunity, blood glucose, and cardiac color sonography. Urine output, lymphocyte ratio, serum albumin, blood calcium, dialysis status, serum urea nitrogen, urine protein, and diabetes significantly correlated with male fecal microbiota composition, whereas only creatinine and 2-h post-prandial blood glucose significantly correlated with female fecal microbiota composition. The top 50 dominant operational taxonomic units showed a stronger correlation with physiological and biochemical indices in samples obtained from females than from males. These differences highlight sex-specific differences in the effectiveness of ESRD prevention and treatments *via* regulating intestinal microbiota.

## Introduction

Chronic kidney disease (CKD) is a global public health problem that affects 10–16% of the adult population worldwide (Meguid El Nahas and Bello, 2005; Barsoum, 2006; Hallan et al., 2006; Coresh et al., 2007; Wen et al., 2008; Stevens et al., 2010; GBD Chronic Kidney Disease Collaboration, 2020). End-stage renal disease (ESRD) requires dialysis or kidney transplantation for survival, which significantly affect patient welfare (Hall, 2021). Moreover, ESRD increases the risk of other diseases, such as cardiovascular diseases (Blacher et al., 1999; London et al., 2003). Although the prevalence of CKD has been relatively stable over the last several years in America, the number of patients with incident ESRD in 2018 was 131,636, which represented an increase of 2.3% compared to 2017 (Johansen et al., 2021). Therefore, exploring the factors and mechanisms that lead to ESRD is of great practical significance for its prevention and clinical treatment.

Gut microbiota is crucial for metabolic, nutritional, physiological, and immunological processes (Bunker et al., 2017; Guldris et al., 2017). The gut microbiota maintains a symbiotic relationship with the host under normal conditions, and it has recently been associated with several diseases, such as inflammatory bowel disease (Caruso et al., 2020), diabetes (Patterson et al., 2016), colorectal cancer (Arthur et al., 2012), and liver cancer (Hartmann and Kronenberg, 2018; Ma et al., 2018). Dysbiosis of gut microbiota contributes to systemic inflammation and accumulation of uremic toxins, which significantly influence the pathophysiology of atherosclerosis and other complications associated with CKD (Guldris et al., 2017). Colon-derived solutes that are normally excreted in the urine accumulate in the plasma when the kidneys fail and may contribute to uremic toxicity (Meyer and Hostetter, 2012). Moreover, ESRD can significantly modify the composition of the gut microbiota in humans (Vaziri et al., 2013).

Diabetes is the leading cause of CKD in developed countries (US Renal Data System, 2011). Type-2 diabetes mellitus (T2DM) has shown a high prevalence rate in developed countries and is an important threat to human health (Bergman, 2007; Runge, 2007; Tönnies et al., 2019). Moreover, the proportion of renal failure attributed to T2DM mellitus is increasing. In the United States, T2DM is the leading cause of ESRD, accounting for 45.9% of the cases of kidney allograft failure from 2011 to 2015 (Alhamad et al., 2019). People with diabetes and CKD have a significantly increased risk of all-cause mortality, cardiovascular mortality, and kidney failure (Fox et al., 2012). Furthermore, the gut microbiota is closely correlated with diabetes (Qin et al., 2012; Patterson et al., 2016; Ni et al., 2021).

The analysis of gender differences in intestinal microbiota in patients with CKD and ESRD is great significance to determine whether gender influence needs to be considered in the treatment of CKD and ESRD, because CKD, ESRD, and intestinal microbiota are closely related to the host immune processes. However, although several studies have demonstrated that intestinal microbiota is associated with diabetes, CKD, and ESRD, it is still not clear whether there are gender differences between these effects. The correlation between intestinal microbiota and other diseases, such as hepatocellular carcinoma (Huang et al., 2018), shows gender differences. Therefore, we speculated that there may also be gender differences in the relationship between intestinal microbiota and ESRD. To test this hypothesis, we compared the differences in the gut microbiota of male and female patients with ESRD caused by IgA nephropathy (ESRD-IgAN) or type-2 diabetes mellitus (ESRD-T2DM) to those of healthy controls (HC) using high-throughput sequencing of the 16S rRNA gene. We also analyzed their correlations based on clinical immune indicators.

## Materials and Methods

### Experimental Design and Sample Collection

The study protocol was approved by the Clinical Research and Application Ethics Committee of the Second Affiliated Hospital of Guangzhou Medical University (approval number: 2021-hs-14) and was performed in accordance with the Clinical Ethics Guidelines and the Declaration of Helsinki and Rules of Good Clinical Practice. Volunteers who agreed to participate in the research project were recruited from inpatients with ESRD and physical examination subjects in the Second Affiliated Hospital of Guangzhou Medical University from October 2020 to March 2021. The inclusion criteria for ESRD-IgAN patients were defined as glomerular filtration rate of less than 20 mL/min/1.73 m^2^ or required dialysis caused by primary IgA nephropathy according to the CKD Epidemiology Collaboration equations (Tent et al., 2010), with the absence of severe psychiatric or psychological disorders. IgA nephropathy was confirmed by biopsy and defined by the finding of dominant or codominant mesangial deposits of IgA. The inclusion criteria for ESRD-T2DM were defined as glomerular filtration rate of less than 20 mL/min/1.73 m^2^ or required dialysis, with absence of severe psychiatric or psychological disorders caused by T2DM. Age-matched volunteers without clinically diagnosable diseases were recruited as HC, and none of the participants received antibiotics or probiotics within 3 months prior to sampling. The ESRD samples were collected according to the time in which the patients who met the inclusion criteria agreed to participate in this research project. Since the patients agreed to participate in this research project was random, the sampling process is also random. The HC subjects were also collected according to the time that they agreed to participate in this research project, while age-unmatched HC subjects were eliminated.

Blood and urine samples were collected according to the clinical diagnostic criteria to determine the physicochemical and immunological indices (Wang et al., 2020). Fecal samples were collected by fecal collectors for microbiota composition analysis (Wang et al., 2020). The estimated glomerular filtration rate (eGFR) was estimated using the Modification of Diet in Renal Disease formula.

### Physiological and Biochemical Indices

The clinical data of the patients were collected, and biochemical or cytological tests of blood and urine specimens were performed as previously described (Kobayashi et al., 2000; Bervoets et al., 2003). Following an overnight fast, venous blood samples were obtained from all subjects and analyzed using standard laboratory methods. A standardized oral glucose tolerance test was performed with 75 g of glucose in 300 ml of water. Hemoglobin A1C (HbA1c) levels were measured by high-performance liquid chromatography using an Adamts H-8160 (Menarini, Florence, Italy). Type 2 diabetes was classified as the use of diabetes medication, Hba1C ≥ 6.5%, oral glucose tolerance test > 11.1 mmol/L after 2 h, or fasting glucose > 7.0 mmol/L. The blood samples were centrifuged, and the obtained plasma was analyzed for creatinine, urea nitrogen, uric acid, albumin, aspartate aminotransferase (AST), and alanine aminotransferase (ALT) using Beckman Coulter AU series instruments (Brea, CA). Cell phenotype was analyzed by staining with fluorochrome-conjugated monoclonal antibodies for the surface markers CD3+, CD4+, and CD8+ (BD Biosciences, Heidelberg, Germany) to determine the lymphocyte subpopulations. The β2-microglobulin, IgA, immunoglobulin G (IgG), and immunoglobulin M (IgM) analyses were performed on a Siemens BNII instrument (Siemens Healthcare Diagnostics, Tarrytown, NY, United States). C-peptide and insulin levels were measured using a Cobas e411 (Roche Diagnostics, Mannheim, Germany) and an AutoDELFIA (Perkin Elmer, Turku, Finland) instrument, respectively. Cardiac ultrasound was used to assess cardiac function and obtain ejection fraction (EF) and fractional shortening (FS) values. In all cases, the samples were blindly analyzed without access to any associated clinical data.

### DNA Extraction and High-Throughput Sequencing of Fecal Microbiota

Microbial DNA was extracted from approximately 0.2 g of fresh fecal sample using a DNeasyPowerSoil Kit (QIAGEN, Germany). The V4–V5 hypervariable region of the 16S rRNA gene was amplified using the universal primers 515F and 909R, as previously described (Huang et al., 2018; Xiang et al., 2018; Ni et al., 2019). Polymerase chain reactions (PCR) were performed in duplicate, and the two PCR products were mixed after amplification, subjected to electrophoresis using 1.5% agarose gel and purified using an AxyPrep DNA gel extraction kit (Axygen, China). The purified DNA was quantified using a Nanodrop 2000 spectrophotometer (Thermo Scientific, United States), pooled together with an equal molar amount from each sample, and sequenced using an Illumina HiSeq system at Guangdong Meilikang Bio-Science Ltd., China.

Raw reads were merged using FLASH 1.2.8 (Magoc and Salzberg, 2011) and processed using quantitative insights into microbial ecology (QIIME) pipeline 1.9.0 (Caporaso et al., 2010) as previously described (Huang et al., 2018; Ni et al., 2019). All merged sequences were trimmed and assigned to each sample according to their barcode sequences. Low-quality and chimera sequences were removed before operational taxonomic unit (OTU) clustering. Then, the high-quality sequences were clustered into OTUs at 97% sequence identity using UPARSE (Edgar, 2013). Taxonomic assignments of each OTU were determined using a ribosomal database project (RDP) classifier (Wang et al., 2007) and the Greengenes gg_13_8_otus dataset.

All DNA datasets were deposited in the National Center for Biotechnology Information (NCBI) Sequence Read Archive database under the accession number PRJNA743980.

### Data Analysis

Principal coordinate analysis (PCoA) was conducted using the QIIME pipeline (Caporaso et al., 2010). Permutational multivariate analysis of variance (PERMANOVA) (Anderson, 2001) was conducted using the vegan package (Dixon, 2003) of R 3.0.1 (R Core Team, 2013). Kruskal–Wallis H tests were conducted using the STAMP software (Parks et al., 2014) to screen the significantly different taxa in each gut microbiota sample. LEfSe analysis (Segata et al., 2011) was conducted to identify the differences in the dominant genera. Statistical significance was set at *P* < 0.05.

## Results

### Physiological and Biochemical Indices of Male and Female Patients

The number of volunteers assigned to the female HC, ESRD-IgAN, and ESRD-T2DM groups, and the male HC, ESRD-IgAN, and ESRD-T2DM groups were 8, 10, 5, 7, 11, and 20, respectively. In the analysis by group, age, and body mass index (BMI) did not present significant differences, indicating that the study groups were homogeneous (Table 1). In general, the height and weight of male volunteers were significantly higher than those of female volunteers (Table 1). Male and female patients with ESRD-IgAN and ESRD-T2DM showed significant increases in urea nitrogen, neutrophil ratio, urine protein, and β2-microglobulin, and a decrease in the lymphocyte ratio (LR). However, there were still inconsistencies between multiple indicators of male and female patients with ESRD-IgAN and ESRD-T2DM. Compared to the control group, male patients with ESRD-IgAN and ESRD-T2DM presented significantly lower urine output, albumin, and aspartate transferase, but significantly higher creatinine levels. However, only those with ESRD-T2DM presented significantly higher glycosylated hemoglobin and fasting serum C-peptide levels, and significantly lower blood calcium (BCa), alanine aminotransferase, and IgM levels. In contrast, female patients showed no significant changes in these indicators (Table 1). Furthermore, female patients with ESRD-IgAN and ESRD-T2DM presented significantly lower hemoglobin levels, and significantly higher 1-h post-prandial blood glucose and 2-h post-prandial blood glucose, but no significant differences were detected for male patients (Table 1).

**TABLE 1:** General characteristics, and physiological and biochemical indices of blood and urine of volunteers.

Item	FHC	FESRD-IgAN	FESRD-T2DM	MHC	MESRD-IgAN	MESRD-T2DM
Sample size	8	10	5	7	11	20
Age	48.63 ± 3.52	47.60 ± 2.90	52.60 ± 3.49	39.86 ± 2.87	42.10 ± 3.21	48.35 ± 1.93
Height (cm)	156.0 ± 1.80^b^	158.1 ± 2.10^b^	160.8 ± 1.50^ab^	172.7 ± 1.86^a^	171.3 ± 1.73^a^	170.5 ± 1.17^a^
Weight (kg)	54.91 ± 2.43^b^	52.84 ± 3.48^b^	53.60 ± 4.93^bc^	73.81 ± 1.88^a^	67.85 ± 3.73^ab^	71.41 ± 2.54^ac^
BMI	22.56 ± 0.88	20.95 ± 0.89	20.70 ± 1.81	24.80 ± 0.83	23.06 ± 1.08	24.50 ± 0.73
**Urine output (mL/d)**	1901.9 ± 208.1^a^	905.0 ± 204.1^ab^	1488.0 ± 156.9^ab^	1941.4 ± 69.7^a^	**581.0 ± 202.6^b^**	**374.9 ± 96.8^b^**
FBG (mmol/L)	4.53 ± 0.14	3.92 ± 0.45	4.96 ± 0.57	4.72 ± 0.21	4.34 ± 0.35	5.44 ± 0.46
**OPBG (mmol/L)**	**7.08 ± 0.77^b^**	10.37 ± 0.56^ab^	**12.30 ± 0.66^a^**	9.65 ± 0.60^ab^	8.80 ± 0.78^ab^	10.97 ± 0.50^a^
**TPBG (mmol/L)**	**5.64 ± 0.37^c^**	9.31 ± 1.07^ab^**^c^**	**13.30 ± 0.88^a^**	6.95 ± 0.79^bc^	8.54 ± 0.40^abc^	11.10 ± 0.75^ab^
**Glycosylated hemoglobin (%)**	5.56 ± 0.05^ab^	5.46 ± 0.08^b^	5.86 ± 0.33^ab^	**5.61 ± 0.04^ab^**	5.30 ± 0.15^b^	**6.44 ± 0.20^a^**
**FSCP (ng/mL)**	3.44 ± 0.32^b^	6.37 ± 0.75^ab^	6.26 ± 0.74^ab^	**4.01 ± 0.41^b^**	8.03 ± 1.13^ab^	**11.61 ± 1.28^a^**
SCPO (ng/mL)	11.26 ± 1.12	15.83 ± 2.43	12.62 ± 2.51	12.00 ± 0.72	16.11 ± 1.93	12.86 ± 1.61
SCPT (ng/mL)	12.46 ± 1.33	21.94 ± 4.03	16.09 ± 4.40	14.49 ± 1.67	20.01 ± 2.89	14.73 ± 1.96
**Creatinine (μ mol/L)**	72.61 ± 3.14^b^	606.92 ± 116.06^ab^	769.56 ± 87.52^ab^	**88.71 ± 3.79^b^**	**938.56 ± 68.19^a^**	**891.23 ± 69.14^a^**
**Urea nitrogen (mmol/L)**	**4.78 ± 0.41^b^**	**24.55 ± 3.01^a^**	**35.39 ± 3.65^a^**	**4.90 ± 0.62^b^**	**20.17 ± 1.83^a^**	**25.39 ± 2.12^a^**
Uric acid (μmol/L)	467.50 ± 144.84	366.90 ± 42.16	423.40 ± 69.45	361.29 ± 19.37	493.70 ± 47.03	445.95 ± 27.59
While blood cell (10^9^/L)	5.86 ± 0.45	6.70 ± 0.54	6.72 ± 0.41	6.73 ± 0.65	7.05 ± 0.40	7.45 ± 0.38
**Neutrophil ratio (%)**	**53.13 ± 2.26^b^**	**67.96 ± 2.19^a^**	67.16 ± 2.09^ab^	**52.97 ± 2.83^b^**	**67.92 ± 2.48^a^**	**67.50 ± 1.65^a^**
**Lymphocyte ratio (%)**	**36.49 ± 2.80^a^**	**21.71 ± 1.35^b^**	**20.82 ± 0.98^b^**	**36.84 ± 2.27^a^**	**19.85 ± 1.83^b^**	**20.56 ± 1.48^b^**
**Hemoglobin (g/L)**	**121.25 ± 4.88^a^**	**91.70 ± 6.57^b^**	**87.60 ± 6.79^b^**	135.57 ± 5.59^a^	102.70 ± 4.97^ab^	106.20 ± 4.38^ab^
RBCC (10^12^/L)	4.16 ± 0.10	3.50 ± 0.25	3.31 ± 0.46	4.64 ± 0.41	3.38 ± 0.14	3.63 ± 0.15
Platelet (10^9^/L)	238.50 ± 33.73	251.60 ± 28.63	185.00 ± 27.74	254.43 ± 29.09	178.70 ± 16.35	210.45 ± 13.00
**Blood calcium (mmol/L)**	2.13 ± 0.23^ab^	2.34 ± 0.06^ab^	2.31 ± 0.06^ab^	**2.49 ± 0.06^a^**	2.23 ± 0.05^ab^	**2.20 ± 0.03^b^**
Blood phosphorus (mmol/L)	6.14 ± 2.28	1.97 ± 0.28	1.83 ± 0.26	2.55 ± 1.27	1.80 ± 0.16	2.06 ± 0.13
Troponin (ng/mL)	1.17 ± 0.75	4.63 ± 1.70	3.48 ± 1.54	1.06 ± 1.04	3.22 ± 1.80	52.52 ± 48.92
**Albumin (g/L)**	40.05 ± 1.36^ab^	38.75 ± 1.88^ab^	35.32 ± 1.22^b^	**46.11 ± 2.15^a^**	**37.53 ± 1.45^b^**	**37.71 ± 0.93^b^**
**Alanine aminotransferase (U/L)**	11.84 ± 1.36^ab^	11.94 ± 4.03^b^	16.64 ± 4.64^ab^	**29.13 ± 6.35^a^**	10.28 ± 1.67^ab^	**10.64 ± 1.25^b^**
**Aspartate transferase (U/L)**	17.59 ± 0.76^ab^	14.41 ± 2.10^ab^	20.28 ± 3.95^ab^	**22.30 ± 1.97^a^**	**10.97 ± 1.31^b^**	**13.91 ± 1.73^b^**
Urine leukocytes (U/L)	9.83 ± 3.44	10.67 ± 3.98	5.00 ± 1.50	13.24 ± 8.49	22.74 ± 16.29	226.92 ± 155.21
**Urine protein**	**0.00 ± 0.00^b^**	**1.30 ± 0.44^a^**	**2.00 ± 0.32^a^**	**0.14 ± 0.14^b^**	**2.10 ± 0.31^a^**	**2.40 ± 0.27^a^**
**β 2-Microglobulin (mg/L)**	**1.55 ± 0.04^b^**	**21.66 ± 2.87^a^**	**21.10 ± 1.98^a^**	**1.67 ± 0.07^b^**	**20.49 ± 2.91^a^**	**24.96 ± 1.47^a^**
CD3+ (%)	75.81 ± 0.69	79.68 ± 1.88	83.42 ± 1.62	75.50 ± 0.58	76.77 ± 2.27	78.51 ± 1.54
CD3 + CD4 + (%)	43.70 ± 0.75	47.21 ± 2.97	55.70 ± 5.06	45.57 ± 1.22	42.36 ± 2.38	44.90 ± 1.82
CD3 + CD8+ (%)	34.58 ± 0.87	28.53 ± 2.21	24.68 ± 4.55	35.56 ± 0.69	29.67 ± 2.33	28.93 ± 1.55
CD19+ (%)	10.26 ± 0.13	8.29 ± 1.13	7.10 ± 1.79	10.74 ± 0.42	11.14 ± 1.51	8.64 ± 0.87
**NK (%)**	**16.78 ± 0.50^a^**	**9.51 ± 1.35^b^**	**8.62 ± 1.60^ab^**	16.56 ± 0.52^a^	10.08 ± 2.22^ab^	10.74 ± 1.63^a^
C3 (g/L)	0.95 ± 0.02	0.69 ± 0.03	0.71 ± 0.04	0.95 ± 0.02	0.81 ± 0.07	2.14 ± 1.30
C4 (g/L)	0.21 ± 0.01	0.19 ± 0.01	0.20 ± 0.01	0.24 ± 0.01	0.19 ± 0.01	0.24 ± 0.01
IgA (g/L)	2.28 ± 0.15	2.80 ± 0.31	2.86 ± 0.57	2.86 ± 0.20	2.15 ± 0.20	2.18 ± 0.24
IgG (g/L)	13.04 ± 0.51^ab^	14.99 ± 1.07^a^	12.87 ± 1.67^ab^	13.06 ± 0.30^ab^	11.02 ± 1.14^b^	11.19 ± 0.65^b^
**IgM (g/L)**	1.33 ± 0.14^a^	1.01 ± 0.08^ab^	1.20 ± 0.40^ab^	**1.26 ± 0.05^a^**	**0.88 ± 0.10^ab^**	**0.70 ± 0.05^b^**
EF (%)	64.38 ± 1.38	67.90 ± 1.49	70.00 ± 1.97	62.00 ± 1.35	64.55 ± 2.83	60.95 ± 2.24
FS (%)	35.13 ± 1.14	38.30 ± 1.17	39.40 ± 1.63	36.14 ± 1.28	35.45 ± 2.04	33.05 ± 1.46

*Data are expressed as mean ± standard error of mean (SEM).FHC, FESRD-IgAN, and FESRD-T2DM indicate female healthy control and patients with end-stage renal disease caused by IgA nephropathy (ESRD-IgAN) or type-2 diabetes mellitus (ESRD-T2DM), respectively. MHC, MESRD-IgAN, and MESRD-T2DM indicate male healthy control and patients with ESRD-IgAN, or ESRD-T2DM, respectively. BMI, body mass index; FBG, fasting blood glucose; OPBG, one-hour post-prandial blood glucose; TPBG, two hours post-prandial blood glucose; FSCP, fasting serum C-peptide; SCPO, serum C-peptide one hour after meal; SCPT, serum C-peptide two hours after meal; RBCC, red blood cell count. NK, nature killer cell; C3, complement 3; C4, complement 4; IgA, immunoglobulin A; IgG, immunoglobulin G; IgM, immunoglobulin M; EF, ejection fraction; FS, fractional shortening. Different lowercases at the upper right of the mean ± standard error showed the statistical significance (p < 0.05).*

*The bold values indicate the indices that showed significant changes in male and female volunteers.*

**FIGURE 1:**
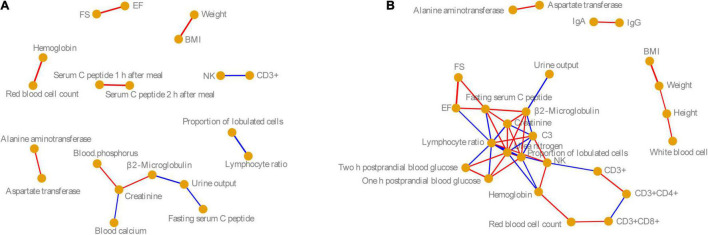
Co-occurrence networks of **(A)** male and **(B)** female physiological and biochemical indices. Nodes indicate the physiological and biochemical indices. Edge width indicates the Pearson correlation. Red and blue edges indicate significantly positive (Pearson correlation coefficient > 0.6 and *P* < 0.05) and significantly negative correlations (Pearson correlation index < −0.6 and *P* < 0.05). BMI, body mass index; NK, nature killer cell; C3, complement 3; IgA, immunoglobulin A; IgG, immunoglobulin G; EF, ejection fraction; FS, fractional shortening.

To analyze the correlation between these physiological and biochemical indices in the male and female volunteers, we calculated the Pearson correlation coefficient of these indices. Based on *P* < 0.05, and Pearson correlation coefficient > 0.6, the physiological and biochemical indices with significant correlation were identified. Our results showed that the correlations between physiological and biochemical indices in men were significantly lower than those in women, especially for indices related to immunity, blood glucose, and cardiac color sonography (Figure 1). However, the significant correlations between physiological and biochemical indices for males and females were consistent. For example, there was a significant positive correlation between FS and EF in both men and women, but for men, these did not significantly positively correlate with fast serum C-peptide as for women. There were significant positive correlations between hemoglobin and red cell count, ALT and AST, and weight and BMI in men and women (Pearson correlation coefficient > 0.6, *P* < 0.05). However, natural killer cells were negatively correlated with CD3+, and the proportion of lobulated cells was negatively correlated with the LR (Pearson correlation coefficient < −0.6, *P* < 0.05; Figure 1).

### Fecal Microbiota Composition of Male and Female Patients

**FIGURE 2:**
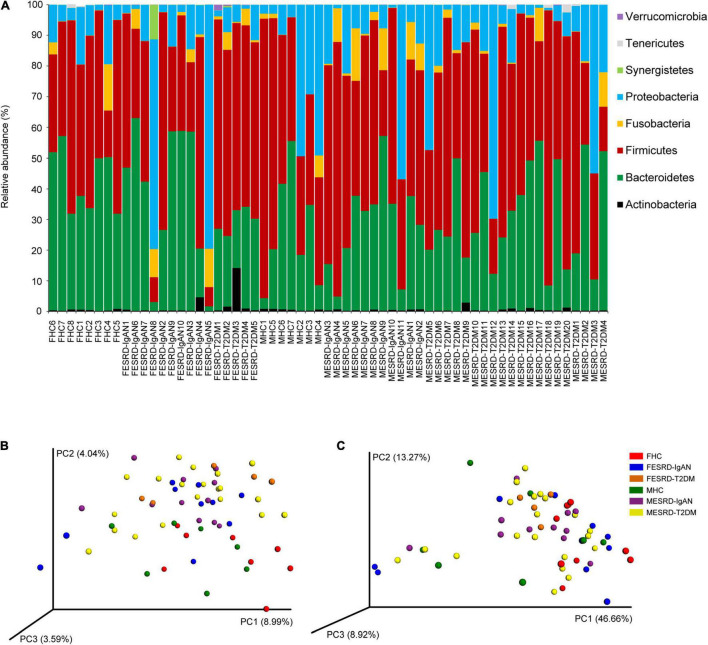
**(A)** Dominant phyla in the fecal microbiota of healthy controls and patients with end-stage renal disease caused by IgA nephropathy or type-2 diabetes mellitus. PCoA profiles based on **(B)** unweighted and **(C)** weighted UniFrac distances of OTU compositions showing changes in fecal microbiota. FHC, FESRD-IgAN, and FESRD-T2DM indicate female healthy control and patients with end-stage renal disease caused by IgA nephropathy (ESRD-IgAN) or type-2 diabetes mellitus (ESRD-T2DM), respectively. MHC, MESRD-IgAN, and MESRD-T2DM indicate male healthy control and patients with ESRD-IgAN, or ESRD-T2DM, respectively.

A total of 2,765,486 (41,275.91 ± 1,680.35) high-quality sequences were obtained from 61 samples. To exclude the influence of sequencing depth on subsequent analysis, 24,573 sequences were randomly selected from each sample for subsequent analysis. Based on 97% similarity, 5,274 OTUs were identified. The sequences (99.995 ± 0.001%) were classified into 18 phyla, in which Firmicutes (48.500 ± 2.533%), Bacteroidetes (31.976 ± 2.183%), Proteobacteria (15.365 ± 2.414%), Fusobacteria (2.946 ± 0.581%), Actinobacteria (0.811 ± 0.239%), Synergistetes (0.215 ± 0.184%), Tenericutes (0.120 ± 0.051%), and Verrucomicrobia (0.050 ± 0.031%) dominated the microbiota (Figure 2A). There was no significant difference in the relative abundance of these dominant phyla between the groups (Kruskal–Wallis rank sum test, *P* > 0.05; Supplementary Figure 1A). Although the alpha diversity in the gut microbiota of CKD patients is commonly significantly altered, our results showed that there was no significant difference in the obtained OTU number, Shannon index, Simpson index, and Chao1 index observed between the groups (Kruskal–Wallis rank sum test, *p* > 0.05; Supplementary Figure 1B). However, PCoA profiles and PERMANOVA indicated that there was a significant difference between the fecal microbiota of male and female patients, including ESRD-IgAN, ESRD-T2DM, and HC groups (PERMANOVA, *F* = 1.762, *P* < 0.001; Figures 2B,C).

**FIGURE 3:**
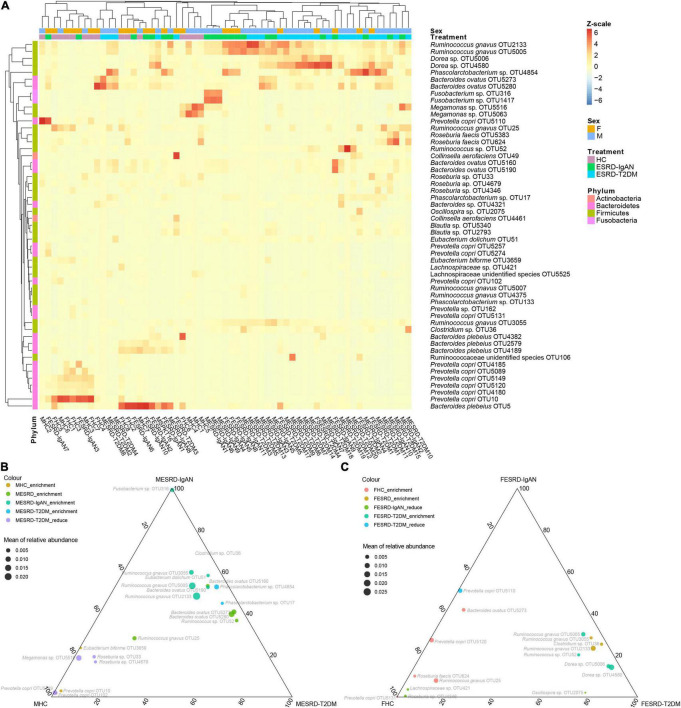
**(A)** Heatmap and **(B,C)** ternary diagram profiles showing the dominant OTU differences in fecal microbiota of male and female healthy controls and patients with end-stage renal disease caused by IgA nephropathy (ESRD-IgAN) or type-2 diabetes mellitus (ESRD-T2DM). FHC, FESRD-IgAN, and FESRD-T2DM indicate female healthy control and patients with end-stage renal disease caused by IgA nephropathy (ESRD-IgAN) or type-2 diabetes mellitus (ESRD-T2DM), respectively. MHC, MESRD-IgAN, and MESRD-T2DM indicate male healthy control and patients with ESRD-IgAN, or ESRD-T2DM, respectively.

To determine which fecal bacteria were different between male and female patients with ESRD-IgAN, ESRD-T2DM, and HC, we used the Kruskal–Wallis H test and Welch’s *post hoc* test to select 53 significantly different OTUs from the 268 dominant OTUs. The obtained OTUs were concentrated in four phyla: Actinobacteria, Bacteroidetes, Firmicutes, and Fusobacteria (Figure 3A). The heatmap profile with cluster analysis also showed that the gender effect appeared weaker than the differences between healthy volunteers and patients with ESRD (Figure 3A). Compared with HC, *Ruminococcus gnavus*, *Ruminococcus* sp., *Eubacterium dolichum*, *Bacteroides ovatus*, and *Phascolarctobacterium* sp. in the fecal microbiota of male ESRD-IgAN or ESRD-T2DM patients were significantly enriched. In contrast, *Megamonas* sp., *Roseburia* sp., and *Eubacterium biforme* were significantly enriched in the fecal microbiota of male HC or significantly decreased in the fecal microbiota of male ESRD-IgAN or ESRD-T2DM patients (Figure 3B). *R. gnavus*, *Clostridium* sp., *Ruminococcus* sp., *Dorea* sp., and *Oscillospira* sp. were significantly enriched in the fecal microbiota of female patients with ESRD-IgAN or ESRD-T2DM, whereas *B. ovatus*, *Prevotella copri*, *Roseburia faecis*, *R. gnavus*, *Lachnospiraceae* sp., and *Roseburia* sp. were significantly enriched in the fecal microbiota of female HC or significantly decreased in the fecal microbiota of female patients with ESRD-IgAN or ESRD-T2DM (Figure 3C).

### Correlation Between Fecal Microbiota and Physiological and Biochemical Indices

**FIGURE 4:**
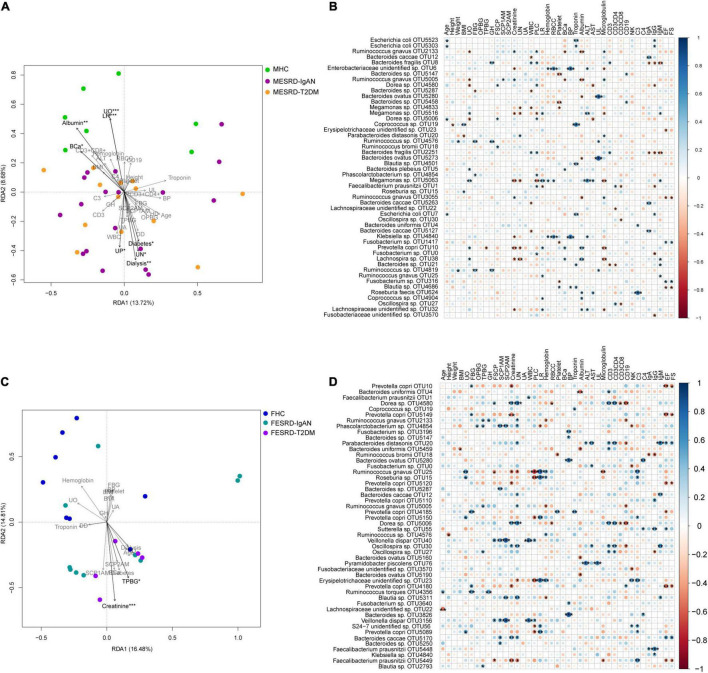
Correlation between physical, blood, and urine indices of volunteers and their fecal microbiota compositions. **(A,C)** Tb-RDA profile showing the correlation between physical, blood, and urine indices of male and female volunteers and their fecal microbiota OTU compositions. **(B,D)** Bubble charts showing the Pearson correlation between physical, blood, and urine indices of male and female volunteers and the top 50 dominant OTUs in their fecal microbiota. FBG, fasting blood glucose; OPBG, one-hour post-prandial blood glucose; TPBG, two hours post-prandial blood glucose; FSCP, fasting serum C-peptide; SCPO, serum C-peptide one hour after meal; SCPT, serum C-peptide two hours after meal; RBCC, red blood cell count. *, *p* < 0.05; **, *p* < 0.01; ***, *p* < 0.001.

To explore the factors responsible for the changes in the fecal microbiota of male and female patients with ESRD-IgAN and ESRD-T2DM, we analyzed the correlation between physical, blood, and urine indices and the fecal microbiota OTU composition using transformation-based redundancy analysis (tb-RDA) and Pearson correlation analysis. The results showed that urine output (UO), LR, serum albumin, BCa, dialysis, urea nitrogen (UN), urine protein (UP), and diabetes were significantly correlated with the male fecal microbiota composition (Figure 4A), whereas only creatinine and Two-h post-prandial blood glucose (TPBG) were significantly correlated with the female fecal microbiota composition (Figure 4C).

In the male samples, *Escherichia coli* was significantly positively correlated with age, troponin, and IgG. Albumin, UO, BCa, and the top 50 dominant OTUs in the fecal samples presented similar trends, as they were significantly negatively correlated with *R. gnavus*, and significantly positively correlated with *P. copri* and *Lachnospira* sp. However, UO was also significantly negatively correlated with *Dorea* sp., and significantly positively correlated with *Megamonas* sp. (Figure 4B). Moreover, *Ruminococcus* sp. was positively correlated with weight, fasting blood glucose, glycosylated hemoglobin (GH), and urine leukocytes (UL). *Klebsiella* sp. showed a similar correlation with an unidentified Enterobacteriaceae OTU, which was positively correlated with red blood cell count, blood phosphorus, and ALT, but negatively correlated with creatinine (Figure 4B). *Megamonas* sp., *P. copri*, and *Lachnospira* sp. were negatively correlated with UN. *Bacteroides fragilis* was positively correlated with white blood cell (WBC), GH, proportion of lobulated cells, and CD3+, but negatively correlated with IgA, IgG, and IgM, whereas *Bacteroides caccae*, *Bacteroides* sp., and *Megamonas* sp. were significantly negatively correlated with WBC count. These results suggest that *Bacteroides* may have a complex function in human immune regulation. *B. ovatus* was positively correlated with UL, and *R. faecis* was positively correlated with complement 3 (C3). The correlation between EF and FS with the top 50 dominant OTUs showed similar trends, that is, they showed significant positive correlation with *Fusobacterium* sp., and significant negative correlation with *R. gnavus* and *Blautia* sp. (Figure 4B).

The top 50 dominant OTUs showed a stronger correlation with the physiological and biochemical indices in the female samples than in the male ones. Although the correlations between several physiological and biochemical indices were consistent for men and women (Figure 1), there were significant differences in the correlations between fecal flora and physiological and biochemical indices. For example, although the correlation between fecal microbiota and EF and FS was consistent for men and women, *Parabacteroides distasonis* and *B. caccae* correlated significantly and positively with EF and FS in female fecal microbiota, while *P. copri* and *Faecalibacterium prausnitzii* showed significant negative correlation (Figure 4D). Only a small number of species had a significant correlation with an index in both male and female samples simultaneously (Figures 4B,D). For women, *P. copri* positively correlated with UO; *Dorea* sp. positively correlated with fasting serum C-peptide; *R. gnavus* and *P. copri* positively correlated with LR, *Coprococcus* sp. positively correlated with troponin, *Oscillospira* sp. positively correlated with CD3+, and *B. ovatus* negatively correlated with albumin. The correlations between other indices and fecal bacteria were completely different (Figures 4B,D). In the top 50 dominant OTUs in female feces, *Ruminococcus torques* significantly positively correlated with age, FBG, and GH. The bacteria with significant positive correlations with SCP1AM and SCP2AM synchronously were *Veillonella dispar* and *Phascolarctobacterium* sp. Moreover, the correlations between female fecal microbiota and creatinine, UN, CD3+, and CD3 + CD4+ were similar, and *Dorea* sp. and *Oscillospira* sp. significantly positively correlated with these four indices (Figure 4D).

## Discussion

Gut inflammation is an important factor in CKD and renal failure, and dysbiosis of the gut microbiota is the main factor leading to intestinal inflammation (Huang et al., 2017). Gut microbes are believed to contribute to uremic syndrome by producing uremic toxins, and additional evidence suggests that the translocation of bacteria and endotoxins (lipopolysaccharides) from the gut to the blood occurs during kidney failure. Consequently, it is plausible to assume that the gut contributes to a chronic inflammatory state in dialysis patients (Kotanko et al., 2006). High serum lipopolysaccharide activity is associated with the development of diabetic nephropathy, obesity, and cardiovascular disease risk (Cani and Delzenne, 2007; Nymark et al., 2009). Moreover, colon-derived solutes that are normally excreted in the urine accumulate in the plasma when the kidneys fail and may also contribute to uremic toxicity (Meyer and Hostetter, 2012). Indoxyl sulfate, a colon-derived uremic solute, has been reported to injure renal tubular cells and may thereby contribute to the progression of renal insufficiency (Niwa, 2010). Therefore, clarifying the correlation between intestinal microbiota and ESRD can provide reference information for the prevention or improvement of ESRD based on the regulation of intestinal microbiota. Our results showed that there are significant differences in the fecal microbiota of male and female HC, ESRD-IgAN, and ESRD-T2DM patients. The dominant bacteria phyla included Actinobacteria, Bacteroidetes, Firmicutes, and Fusobacteria, and the relative abundances of these phyla were not significantly different between male and female HC, ESRD-IgAN, and ESRD-T2DM patients (Kruskal–Wallis rank sum test, *P* > 0.05). Furthermore, the observed bacteria mainly included *Ruminococcus*, *Eubacterium*, *Bacteroides*, *Clostridium*, *Dorea*, *Roseburia*, *Lachnospiraceae*, *Phascolarctobacterium*, and *Megamonas*, which are closely related to human metabolism and immunity, and demonstrate differences between the host sex (Figures 3B,C). Although these bacteria are closely related to human metabolism and immunity, their differences between the host sex seem to be caused by the metabolic differences between females and males. For instance, females have a higher percent body fat than males and females having lower glomerular filtration and activity of uridine diphosphate glucuronosyltransferase enzyme (Anderson, 2008). These differences may lead to differences in metabolism and response to abnormal renal function between females and males, such as creatinine, urea nitrogen, neutrophil ratio, hemoglobin, and aspartate transferase (Table 1), which indirectly resulted differences in gut microbiota, and the differences of gut microbiota in response to abnormal renal function.

Clinical markers related to renal function, including blood urea nitrogen (BUN), serum creatinine (SCr), and serum uric acid (SUA), are commonly used to discriminate between CKD and control cohorts (Ye et al., 2018). ESRD patients usually present with significantly increased plasma concentrations of creatinine and urea nitrogen (Vaziri et al., 2013). Our results showed that urea nitrogen, neutrophil ratio, and urine protein were significantly increased in male and female ESRD-IgAN and ESRD-T2DM patients, whereas the LR was significantly reduced (Table 1). Serum creatinine significantly increased in male ESRD-IgAN and ESRD-T2DM patients. In female ESRD-IgAN and ESRD-T2DM patients, despite the trend of increasing serum creatinine, no significant increase was detected because the intra-group differences were exceedingly large (Table 1). Considering that serum creatinine and urea nitrogen were significantly increased in ESRD patients, and many fecal microbial species significantly correlated with serum creatinine and urea nitrogen (Figure 4), these fecal microbial species may also be related to ESRD. Although their causal relationship and interactions were not clear, the results suggest that the manipulation of gut microbiota can be used as an auxiliary treatment for end-stage renal diseases.

Sexual dimorphisms in anatomical, physiological and behavioral traits are characteristics of many vertebrate species, including human (Ober et al., 2008). Gender differences in the intestinal microbiota also have been reported in many metabolic diseases and malignant tumors (Ba et al., 2017; Bridgewater et al., 2017; Huang et al., 2018; Razavi et al., 2019). Our results showed that there are gender differences in both physiological and biochemical indices and intestinal microbiota composition, especially in the correlation between intestinal microbiota and physiological and biochemical indices (Figure 4). For example, although serum urea nitrogen significantly increased in both male and female patients (Table 1), the bacteria significantly positively correlated with serum urea nitrogen in males was *P. distasonis*, whereas those significantly negatively correlated were *Megamonas* sp., *Fusobacterium* sp., and *Lachnospira* sp. In contrast, the bacteria significantly positively correlated with female serum urea nitrogen were *Dorea* sp., *Ruminococcus* sp., *Phascolarctobacterium* sp., *P. distasonis*, *B. caccae*, *R. gnavus*, *Oscillospira* sp., and *Blautia* sp., and the significantly negatively correlated bacteria were *R. gnavus*, *F. prausnitzii*, and an unidentified bacterium in Erysipelotrichaceae (Figure 4). In addition, the serum immunological indices significantly associated with the top 50 dominant OTUs in the fecal microbiota of men were significantly lower than those in women (Figure 4). These differences may lead to gender differences in the effect of ESRD prevention and treatment through the regulation of intestinal microbiota, which is a clinical issue worthy of consideration. Moreover, because the gut microbiota participates in or affects the metabolic process of drugs (Zimmermann et al., 2019), the impact of sex differences in gut microbiota on the drugs used to treat ESRD should also be paid attention.

Small sample sizes usually lead to a reduced detection efficiency of positive statistical results, especially in cases of clinical interference by a variety of influencing factors. This was a limitation of the present study. Another limitation was that we only analyzed the differences in intestinal microbiota in patients of different sexes with ESRD-IgAN and ESRD-T2DM and their correlation with physiological and biochemical indicators of patients, but we did not further verify this. Although it is relatively difficult to verify the results of our study in a clinic, the results can be verified by tracking the changes in physiological and biochemical indices and intestinal microbiota during long-term treatments. Moreover, accumulation of bacterial uremic retention solutes, such as indoxyl sulfate and p-cresyl sulfate, is associated with CKD (Schepers et al., 2007; Ellis et al., 2016; Chen et al., 2019). Analyzing the relationship between the gut microbiota alteration caused by different factors and the production of these bacterial uremic retention solutions, as well as the relationship between the accumulation of these bacterial uremic retention solutions and the development of CKD, well assist us to deeply understand the role of gut microbiota in the development of CKD, and may provide reference for the prevention of CKD by regulating gut microbiota. Unfortunately, we did not determine these bacterial uremic retention solutions in this study.

In conclusion, our results demonstrate the gender differences in both physiological and biochemical indices and intestinal microbiota composition, especially in the correlations between them. These differences may lead to gender differences in the effect of ESRD prevention and treatment through the regulation of intestinal microbiota, which is a clinical issue worthy of consideration.

## Data Availability Statement

The datasets presented in this study can be found in online repositories. The names of the repository/repositories and accession number(s) can be found below: https://www.ncbi.nlm.nih.gov/, PRJNA743980.

## Ethics Statement

The studies involving human participants were reviewed and approved by the Clinical Research and Application Ethics Committee of the Second Affiliated Hospital of Guangzhou Medical University (approval number: 2021-hs-14). The patients/participants provided their written informed consent to participate in this study.

## Author Contributions

PZ: conception and design. ZC, JM, and WZ: administrative support. ZC, JF, LZ, and GL: provision of study materials or patients. XL, LX, LuL, YX, LiL, TZ, JW, HX, and RC: collection and assembly of data. PZ and ZC: data analysis and interpretation. PZ: manuscript writing. All authors: final approval of manuscript.

## Conflict of Interest

The authors declare that the research was conducted in the absence of any commercial or financial relationships that could be construed as a potential conflict of interest.

## Publisher’s Note

All claims expressed in this article are solely those of the authors and do not necessarily represent those of their affiliated organizations, or those of the publisher, the editors and the reviewers. Any product that may be evaluated in this article, or claim that may be made by its manufacturer, is not guaranteed or endorsed by the publisher.

## Supplementary Material

Supplementary Figure 1 Relative abundances of dominant phyla in the fecal microbiota of healthy controls and patients with end-stage renal disease caused by IgA nephropathy or type-2 diabetes mellitus **(A)** and commonly used alpha diversity indices of the fecal microbiota **(B)**.

## References

[B1] Alhamad T. Kunjal R. Wellen J. Brennan D. C. Wiseman A. Ruano K. (2019). Three-month pancreas graft function significantly influences survival following simultaneous pancreas-kidney transplantation in type 2 diabetes patients. *Am. J. Transplant.* 20 788–796. 10.1111/ajt.15615 31553823

[B2] Anderson G. D. (2008). Gender differences in pharmacological response. *Int. Rev. Neurobiol.* 83 1–10. 10.1016/S0074-7742(08)00001-9 18929073

[B3] Anderson M. J. (2001). A new method for non-parametric multivariate analysis of variance. *Austral Ecol.* 26 32–46. 10.1111/j.1442-9993.2001.01070.pp.x

[B4] Arthur J. C. Perez-Chanona E. Mühlbauer M. Tomkovich S. Uronis J. M. Fan T.-J. (2012). Intestinal inflammation targets cancer-inducing activity of the microbiota. *Science* 338 120–123. 10.1126/science.1224820 22903521 PMC3645302

[B5] Ba Q. Li M. Chen P. Huang C. Duan X. Lu L. (2017). Sex-dependent effects of cadmium exposure in early life on gut microbiota and fat accumulation in mice. *Environ. Health Perspect.* 125 437–446. 10.1289/EHP360 27634282 PMC5332190

[B6] Barsoum R. S. (2006). Chronic kidney disease in the developing world. *N. Engl. J. Med.* 354 997–999. 10.1056/NEJMp058318 16525136

[B7] Bergman S. A. (2007). Perioperative management of the diabetic patient. *Oral Surg. Oral Med. Oral Pahtol. Oral Radiol. Endod.* 103 731–737. 10.1016/j.tripleo.2006.11.029 17376713

[B8] Bervoets A. R. J. Spasovski G. B. Behets G. J. Dams G. Polenakovic M. H. Zafirovska K. (2003). Useful biochemical markers for diagnosing renal osteodystrophy in predialysis end-stage renal failure patients. *Am. J. Kidney Dis.* 41 997–1007. 10.1016/s0272-6386(03)00197-5 12722034

[B9] Blacher J. Guerin A. P. Pannier B. Marchais S. J. Safar M. E. London G. M. (1999). Impact of aortic stiffness on survival in end-stage renal disease. *Circulation* 99 2434–2439. 10318666 10.1161/01.cir.99.18.2434

[B10] Bridgewater L. C. Zhang C. Wu Y. Hu W. Zhang Q. Wang J. (2017). Gender-based differences in host behavior and gut microbiota composition in response to high fat diet and stress in a mouse model. *Sci. Rep.* 7:10776. 10.1038/s41598-017-11069-4 28883460 PMC5589737

[B11] Bunker J. J. Erickson S. A. Flynn T. M. Henry C. Koval J. Meisel M. (2017). Natural polyreactive IgA antibodies coat the intestinal microbiota. *Science* 358:eaan6619. 10.1126/science.aan6619 28971969 PMC5790183

[B12] Cani P. D. Delzenne N. M. (2007). Gut microflora as a target for energy and metabolic homeostasis. *Curr. Opin. Clin. Nutr. Metab. Care* 10 729–734. 10.1097/MCO.0b013e3282efdebb 18089955

[B13] Caporaso J. G. Kuczynski J. Stombaugh J. Bittinger K. Bushman F. D. Costello E. K. (2010). QIIME allows analysis of high-throughput community sequencing data. *Nat. Methods* 7 335–336. 10.1038/nmeth.f.303 20383131 PMC3156573

[B14] Caruso R. Lo B. C. Núñez G. (2020). Host-microbiota interactions in inflammatory bowel disease. *Nat. Rev. Immunol.* 20 411–426. 10.1038/s41577-019-0268-7 32005980

[B15] Chen Y. Y. Chen D. Q. Chen L. Liu J. R. Vaziri N. D. Guo Y. (2019). Microbiome-metabolome reveals the contribution of gut-kidney axis on kidney disease. *J. Transl. Med.* 17:5. 10.1186/s12967-018-1756-4 30602367 PMC6317198

[B16] Coresh J. Selvin E. Stevens L. A. Manzi J. Kusek J. W. Eggers P. (2007). Prevalence of chronic kidney disease in the United States. *JAMA* 298 2038–2047. 10.1001/jama.298.17.2038 17986697

[B17] Dixon P. (2003). VEGAN, a package of R functions for community ecology. *J. Veg. Sci.* 14 927–930. 10.1111/j.1654-1103.2003.tb02228.x

[B18] Edgar R. C. (2013). UPARSE: highly accurate OTU sequences from microbial amplicon reads. *Nat. Methods* 10 996–998. 10.1038/nmeth.2604 23955772

[B19] Ellis R. J. Small D. M. Vesey D. A. Johnson D. W. Francis R. Vitetta L. (2016). Indoxyl sulphate and kidney disease: causes, consequences and interventions. *Nephrology* 21 170–177. 10.1111/nep.12580 26239363

[B20] Fox C. S. Matsushita K. Woodward M. Bilo H. J. G. Chalmers J. Lambers Heerspink H. J. (2012). Associations of kidney disease measures with mortality and end-stage renal disease in individuals with and without diabetes: a meta-analysis. *Lancet* 380 1662–1673. 10.1016/S0140-6736(12)61350-6 23013602 PMC3771350

[B21] GBD Chronic Kidney Disease Collaboration (2020). Global, regional, and national burden of chronic kidney disease, 1990-2017: a systematic analysis for the Global Burden of Disease Study 2017. *Lancet* 395 709–733. 10.1016/S0140-6736(20)30045-3 32061315 PMC7049905

[B22] Guldris S. C. Parra E. G. Amenós A. C. (2017). Gut microbiota in chronic kidney disease. *Nefrologia* 37 9–19. 10.1016/j.nefro.2016.05.008 27553986

[B23] Hall R. K. (2021). Prioritizing the quality of life of older adults with kidney disease. *Nat. Rev. Nephrol.* 17 149–150. 10.1038/s41581-021-00397-4 33473235

[B24] Hallan S. I. Coresh J. Astor B. C. Åsberg A. Powe N. R. Romundstad S. (2006). International comparison of the relationship of chronic kidney disease prevalence and ESRD risk. *J. Am. Soc. Nephrol.* 17 2275–2284. 10.1681/ASN.2005121273 16790511

[B25] Hartmann N. Kronenberg M. (2018). Cancer immunity thwarted by the microbiome. *Science* 360 858–859. 10.1126/science.aat8289 29798871

[B26] Huang R. Li T. Ni J. Bai X. Gao Y. Li Y. (2018). Different sex-based responses of gut microbiota during the development of hepatocellular carcinoma in liver-specific Tsc1-knockout mice. *Front. Microbiol.* 9:1008. 10.3389/fmicb.2018.01008 29867896 PMC5964185

[B27] Huang W. Zhou L. Guo H. Xu Y. Xu Y. (2017). The role of short-chain fatty acids in kidney injury induced by gut-derived inflammatory response. *Metabolism* 68 20–30. 10.1016/j.metabol.2016.11.006 28183450

[B28] Johansen K. L. Chertow G. M. Foley R. N. Gilbertson D. T. Herzog C. A. Ishani A. (2021). US Renal Data System 2020 Annual Data Report: epidemiology of kidney disease in the United States. *Am. J. Kidney Dis.* 77 Svii-Sviii, S1-S597. 10.1053/j.ajkd.2021.01.002 PMC8148988 33752804

[B29] Kobayashi S. Maejima S. Ikeda T. Nagase M. (2000). Impact of dialysis therapy on insulin resistance in end-stage renal disease: comparison of haemodialysis and continuous ambulatory peritoneal dialysis. *Nephrol. Dial. Transplant.* 15 65–70. 10.1093/ndt/15.1.65 10607769

[B30] Kotanko P. Carter M. Levin N. W. (2006). Intestinal bacterial microflora – a potential source of chronic inflammation in patients with chronic kidney disease. *Nephrol. Dial. Transplant.* 21 2057–2060. 10.1093/ndt/gfl281 16762961

[B31] London G. M. Guérin A. P. Marchais S. J. Métivier F. Pannier B. Adda H. (2003). Arterial media calcification in end-stage renal disease: impact on all-cause and cardiovascular mortality. *Nephrol. Dial. Transplant.* 18 1731–1740. 10.1093/ndt/gfg414 12937218

[B32] Ma C. Han M. Heinrich B. Fu Q. Zhang Q. Sandhu M. (2018). Gut microbiome-mediated bile acid metabolism regulates liver cancer via NKT cells. *Science* 876:eaan5931. 10.1126/science.aan5931 29798856 PMC6407885

[B33] Magoc T. Salzberg S. L. (2011). FLASH: fast length adjustment of short reads to improve genome assemblies. *Bioinformatics* 27 2957–2963. 10.1093/bioinformatics/btr507 21903629 PMC3198573

[B34] Meguid El Nahas A. Bello A. K. (2005). Chronic kidney disease: the global challenge. *Lancet* 365 331–340. 10.1016/S0140-6736(05)17789-7 15664230

[B35] Meyer T. W. Hostetter T. H. (2012). Uremic solutes from colon microbes. *Kidney Int.* 81 949–954. 10.1038/ki.2011.504 22318422

[B36] Ni J. Fu C. Huang R. Li Z. Li S. Cao P. (2021). Metabolic syndrome cannot mask the changes of faecal microbiota compositions caused by primary hepatocellular carcinoma. *Lett. Appl. Microbiol.* 73 73–80. 10.1111/lam.13477 33768575

[B37] Ni J. Huang R. Zhou H. Xu X. Li Y. Cao P. (2019). Analysis of the relationship between the degree of dysbiosis in gut microbiota and prognosis at different stages of primary hepatocellular carcinoma. *Front. Microbiol.* 10:1458. 10.3389/fimcb.2019.01458 31293562 PMC6603198

[B38] Niwa T. (2010). Indoxyl sulfate is a nephron-vascular toxin. *J. Ren. Nutr.* 20 S2–S6. 20797565 10.1053/j.jrn.2010.05.002

[B39] Nymark M. Pussinen P. J. Tuomainen A. M. Forsblom C. Groop P. H. Lehto M. (2009). Serum lipopolysaccharide activity is associated with the progrwssion of kidney disease in Finnish patients with type 1 diabetes. *Diabetes Care* 32 1689–1693. 10.2337/dc09-0467 19502539 PMC2732155

[B40] Ober C. Liosel D. A. Gilad Y. (2008). Sex-specific genetic architecture of human disease. *Nat. Rev. Genetics* 9 911–922. 10.1038/nrg2415 19002143 PMC2694620

[B41] Parks D. H. Tyson G. W. Hugenholtz P. Beiko R. G. (2014). STAMP: statistical analysis of taxonomic and functional profiles. *Bioinformatics* 30, 3123–3124. 10.1093/bioinformatics/btu494 25061070 PMC4609014

[B42] Patterson E. Ryan P. M. Cryan J. F. Dinan T. G. Ross R. P. Fitzgerald G. F. (2016). Gut microbiota, obesity and diabetes. *Postgrad. Med. J.* 92 286–300. 10.1136/postgradmedj-2015-133285 26912499

[B43] Qin J. Li Y. Cai Z. Li S. Zhu J. Zhang F. (2012). A metagenome-wide association study of gut microbiota in type 2 diabetes. *Nature* 490 55–60. 10.1038/nature11450 23023125

[B44] Razavi A. C. Potts K. S. Kelly T. N. Bazzano L. A. (2019). Sex, gut microbiome, and cardiovascular disease risk. *Biol. Sex Differ.* 10:29. 10.1186/s13293-019-0240-z 31182162 PMC6558780

[B45] R Core Team (2013). *R: A Language and Environment for Statistical Computing.* Vienna: R Foundation for Statistical Computing. Available online at: http://www.R-project.org/

[B46] Runge C. F. (2007). Economic consequences of the obese. *Diabetes* 56 2668–2672. 10.2337/db07-0633 17601989

[B47] Schepers E. Meert N. Glorieux G. Goeman J. Van der Eycken J. Vanholder R. (2007). P-cresylsulphate, the main in vivo metabolite of p-cresol, activates leucocyte free radical production. *Nephrol. Dial. Transplant.* 22 592–596. 10.1093/ndt/gf1584 17040995

[B48] Segata N. Izard J. Waldron L. Gevers D. Miropolsky L. Garrett W. S. (2011). Metagenomic biomarker discovery and explanation. *Genome Biol.* 12:R60. 10.1186/gb-2011-12-6-r60 21702898 PMC3218848

[B49] Stevens L. A. Visvanathan G. Weiner D. E. (2010). CKD and ESRD in the elderly: current prevalence, future projections, and clinical significance. *Adv. Chronic Kidney Dis.* 17 293–301. 10.1053/j.ackd.2010.03.010 20610356 PMC3160131

[B50] Tent H. Rook M. Stevens L. A. van Son W. J. van Pelt J. Hofker H. S. (2010). Renal function equations before and after living kidney donation: a within-individual comparison of performance at different levels of renal functions. *Clin. J. Am. Soc. Nephrol.* 5 1960–1968. 10.2215/CJN.08761209 20616162 PMC3001772

[B51] Tönnies T. Röckl S. Hoyer A. Heidemann C. Baumert J. Du Y. (2019). Projected number of people with diagnosed type 2 diabetes in Germany in 2040. *Diabet. Med.* 36 1217–1225. 10.1111/dme.13902 30659656

[B52] US Renal Data System (2011). *USRDS 2011 Annual Data Report: Atlas of Chronic Kidney Disease and End-Stage Renal Disease in the United States.* Bethesda MD: National Institutes of Health, National Institute of Diabetes and Digestive and Kidney Diseases.

[B53] Vaziri N. D. Wong J. Pahl M. Piceno Y. M. Yuan J. DeSantis T. Z. (2013). Chronic kidney disease alters intestinal microbial flora. *Kidney Int.* 83 308–315. 10.1038/ki.2012.345 22992469

[B54] Wang Q. Garrity G. M. Tiedje J. M. Cole J. R. (2007). Naïve Bayesian classifier for rapid assignment of rRNA sequences into the new bacterial taxonomy. *Appl. Environ. Microbiol.* 73 5261–5267. 10.1128/AEM.00062-07 17586664 PMC1950982

[B55] Wang X. Yang S. Li S. Zhao L. Hao Y. Qin J. (2020). Aberrant gut microbiota alters host metabolome and impacts renal failure in humans and rodents. *Gut* 69 2131–2142. 10.1136/gutjnl-2019-319766 32241904 PMC7677483

[B56] Wen C. P. Cheng T. Y. Tsai M. K. Chang Y. C. Chan H. T. Tsai S. P. (2008). All-cause mortality attributable to chronic kidney disease: a prospective cohort study based on 462293 adults in Taiwan. *Lancet* 371 2173–2182. 10.1016/S0140-6736(08)60952-6 18586172

[B57] Xiang J. He T. Wang P. Xie M. Xiang J. Ni J. (2018). Opportunistic pathogens are abundant in the gut of cultured giant spiny frog (Paa spinosa). *Aquac. Res.* 49 2033–2041. 10.1111/are.13660

[B58] Ye G. Zhou M. Yu L. Ye J. Yao L. Shi L. (2018). Gut microbiota in renal transplant recipients, patients with chronic kidney disease and healthy subjects. *J. South Med. Univ.* 38 1401–1408. 10.12122/j.issn.1673-4254.2018.12.01 30613005 PMC6744200

[B59] Zimmermann M. Zimmermann-Kogadeeva M. Wegmann R. Goodman A. L. (2019). Separating host and microbiome contributions to drug pharmacokinetics and toxicity. *Science* 363:eaat9931. 10.1126/science.aat9931 30733391 PMC6533120

