# Supplementary figures and images for: Sex Differences in Fecal Microbiota Correlation With Physiological and Biochemical Indices Associated With End-Stage Renal Disease Caused by Immunoglobulin a Nephropathy or Diabetes
Source: Front Microbiol. 2021 Nov 26;12:752393. doi: 10.3389/fmicb.2021.752393 (PMC8661007; doi:10.3389/fmicb.2021.752393)

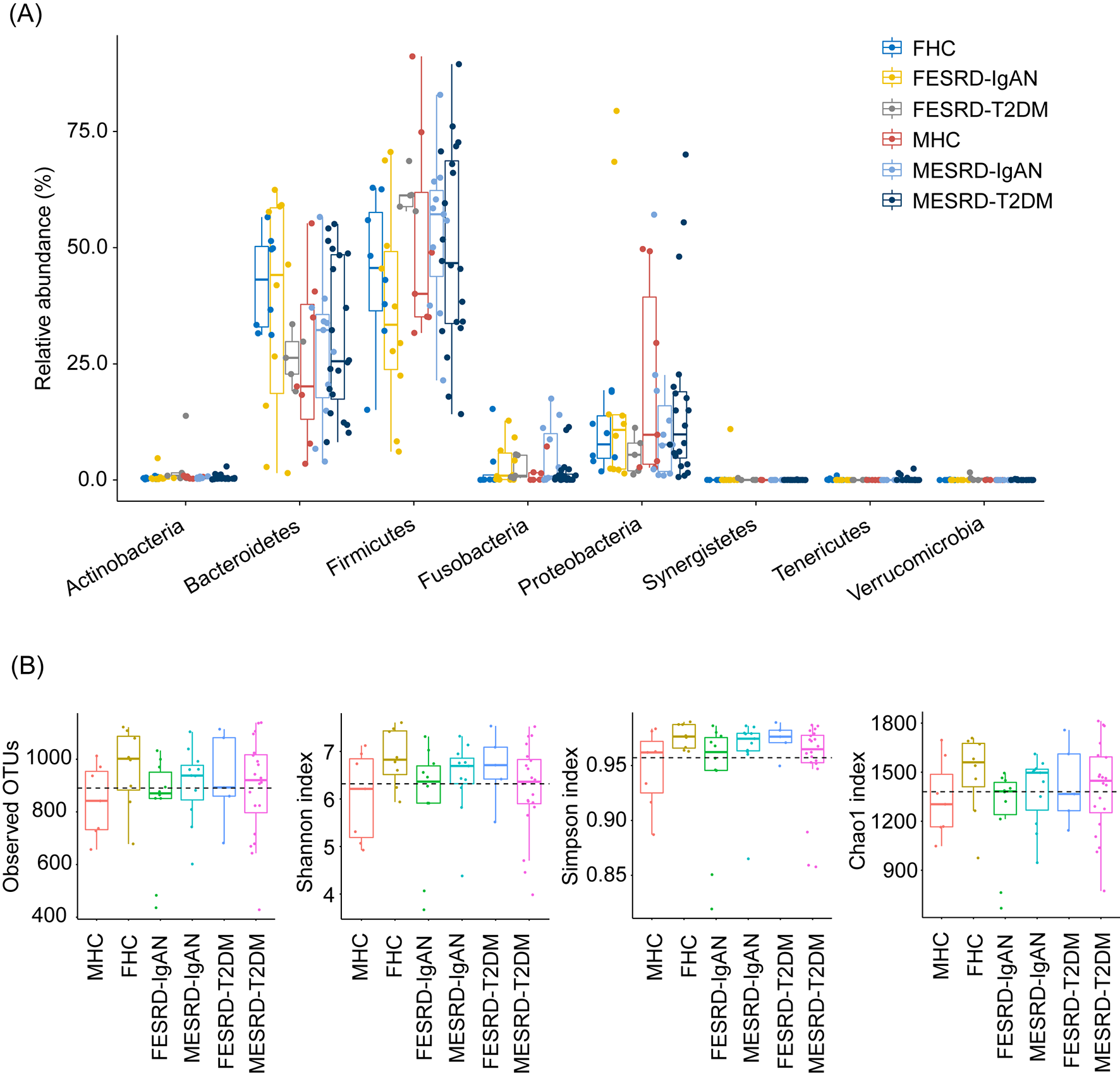

Supplement: Supplementary Figure 1 — Relative abundances of dominant phyla in the fecal microbiota of healthy controls and patients with end-stage renal disease caused by IgA nephropathy or type-2 diabetes mellitus (A) and commonly used alpha diversity indices of the fecal microbiota (B). [file Image_1.TIF]
